# Telemedicine in Chronic Wound Management: Systematic Review And Meta-Analysis

**DOI:** 10.2196/15574

**Published:** 2020-06-25

**Authors:** Lihong Chen, Lihui Cheng, Wei Gao, Dawei Chen, Chun Wang, Xingwu Ran

**Affiliations:** 1 Diabetic Foot Care Center, Department of Endocrinology and Metabolism West China Hospital Sichuan University Chengdu China; 2 Department of Clinical Laboratory Nuclear Industry 416 Hospital, the 2nd Affiliated Hospital of Chengdu Medical College Chengdu China; 3 School of Clinical Medicine Southwest Medical University Luzhou China

**Keywords:** telemedicine, wounds and injuries, wound healing, meta-analysis

## Abstract

**Background:**

Chronic wounds have been a great burden to patients and the health care system. The popularity of the internet and smart devices, such as mobile phones and tablets, has made it possible to adopt telemedicine (TM) to improve the management of chronic wounds. However, studies conducted by different researchers have reported contradictory results on the effect of TM on chronic wound management.

**Objective:**

The aim of this work was to evaluate the efficacy and safety of TM in chronic wound management.

**Methods:**

We systematically searched multiple electronic databases (MEDLINE, EMBASE, and Cochrane Central Register of Controlled Trials [CENTRAL]) to identify eligible studies published from inception to June 12, 2019. Inclusion criteria were randomized controlled trials (RCTs) and interventional cohort studies that investigated the use of TM in chronic wound management. RCT and observational data were analyzed separately. A meta-analysis and qualitative analysis were conducted to estimate endpoints.

**Results:**

A total of 6 RCTs and 6 cohort studies including 3913 patients were included. Of these, 4 studies used tablets or mobile phones programmed with apps, such as Skype and specialized interactive systems, whereas the remaining 8 studies used email, telephone, and videoconferencing to facilitate the implementation of TM using a specialized system. Efficacy outcomes in RCTs showed no significant differences in wound healing (hazard ratio [HR] 1.16, 95% CI 0.96-1.39; *P*=.13), and wound healing around 1 year (risk ratio [RR] 1.05, 95% CI 0.89-1.23; *P*=.15). Noninferiority criteria of TM were met. A decreased risk of amputation in patients receiving TM was revealed (RR 0.45, 95% CI 0.29-0.71; *P*=.001). The result of cohort studies showed that TM was more effective than standard care (HR 1.74, 95% CI 1.43-2.12; *P*<.001), whereas the outcome efficacy RR of wound healing around 1 year (RR 1.21, 95% CI 0.96-1.53; *P*=.56) and 3 months (RR 1.24, 95% CI 0.47-3.3; *P*=.67) was not significantly different between TM and standard care. Noninferiority criteria of TM were met for wound healing around 1 year in cohort studies.

**Conclusions:**

Currently available evidence suggests that TM seems to have similar efficacy and safety, and met noninferiority criteria with conventional standard care of chronic wounds. Large-scale, well-designed RCTs are warranted.

## Introduction

A chronic wound is defined as a break in the skin that failed to progress through a normal sequence of repair in 4-8 weeks [1]. Venous stasis ulcers, arterial ulcers, neuropathic ulcers, pressure ulcers, diabetic foot ulcers, and ulcers due to malignancy are examples of chronic wounds. This has become a great challenge and burden to patients, health care professionals, and the health care systems. There are over 6 million chronic wounds in the United States, which accounts for an estimated US $25 billion annually in the US health care costs [2]. Thus, there is huge pressure on the health care system to develop cost-effective wound management practices.

Telemedicine (TM) is the use of telecommunication technologies to provide remote clinical services to patients to improve the quality of individual treatments. The concept of TM dates back to the 19th century. It was practiced via telegraph, telephone, and radio before the internet existed [3,4]. With the ascent of the information age, networks and smartphones have shown great potential in providing remote clinical services. It has been widely used in various areas of health care such as heart conditions [5], diabetes mellitus care [6], and management of individuals with chronic obstructive pulmonary disease [7].

Within wound care, TM could support access to expertise in remote areas to improve management of chronic wounds in geographically challenging environments [8,9]. Because of the lack of wound care specialists and the financial pressure on health care agencies, TM could be introduced as an alternative solution to support task shifting of experts from hospitals to underserved populations or rural areas. This could contribute to a reduction in the number of consultations and associated transportation costs [10]. It is also important to realize these goals without impinging on the quality of care. Furthermore, the development of mobile phone apps make it convenient to implement the TM for diabetic foot ulcers [11,12].

Findings from qualitative studies show positive results with several systematic reviews in recent years being published [13-16]. With the convenience and accuracy of communication in the information age, there has been a growing interest in the management of chronic wound via TM. However, the impact of TM on wound healing was inconsistent, with 2 reports indicating positive results [17,18], 1 negative [19], and 3 showing no change [20-22]. In terms of mortality and amputation, 2 RCTs revealed inconsistent results [20,22]. Thus, there is a rational to conduct a systematic review to clarify the effect of TM on chronic wounds.

Our objective was to conduct a systematic review and meta-analysis of randomized and interventional cohort studies. We sought to investigate whether TM follow-up in community care in collaboration with specialists in wound center is noninferior to the conventional standard care of chronic wounds.

## Methods

### Eligible Criteria

The systematic review and meta-analysis were conducted in accordance with recommendations by the Cochrane Collaboration [23] and the PRISMA statement [24]. Inclusion criteria were randomized controlled trials (RCT) and interventional cohort studies that investigated the use of TM follow-up in the community care in collaboration with specialists in wound centers with a comparator of no TM. Only articles that investigated chronic wounds, such as diabetic foot ulcers, stasis ulcer, pressure ulcers, and nonhealing surgical wound, were included. Exclusion criteria included case reports, editorials, letters, animal studies, case–control studies, and self-control studies. Non-English articles were excluded.

### Search Strategy and Study Selection

A systematic search of databases (PubMed/MEDLINE, EMBASE, and Cochrane Central Register of Controlled Trials [CENTRAL]) was conducted to identify eligible studies published from inception to June 12, 2019. The reference lists of all identified articles and reviews were searched for potentially eligible studies. Only published articles were included. The search strategy is available in Multimedia Appendix 1. Two investigators selected studies independently (LC1 and WG). Disagreements were resolved by discussion with a consensus decision or by the decision of another author (XR). In case of duplicates, or multiple reports of a primary study, only the report with the most complete data set was included.

### Evaluation of Bias

The bias of RCTs included in the systematic review was assessed using the Cochrane’s tool for assessing risk of bias [23], whereas the risk of bias in interventional cohort studies was assessed using ROBINS-I [25]. The evaluation was made by 2 independent assessors to ensure validity (LC1 and LC2). Disagreements were resolved by consensus.

### Data Extraction

Data extraction was performed independently by two reviewers (LC1 and LC2). Any discrepancies were resolved by discussion or by a third investigator (XR). All studies included in the meta-analysis had to be either RCTs or cohort studies. The prespecified primary outcomes were wound healing; the secondary outcomes were all-cause mortality, amputation, number of consultations, and patient experience.

### Statistical Analysis

A meta-analysis was performed using Stata 12.0 (Stata Corp) and RevMan 5.3 (The Nordic Cochrane Centre, The Cochrane Collaboration). Hazard ratio (HR) and associated statistics were either extracted directly from articles or estimated from Kaplan–Meier curves [26]. Where sufficient data were available, a meta-analysis was conducted with a primary outcome of wound healing. The HRs for the unhealed wounds, with a value >1 favoring TM, were combined using a random-effect model (DerSimonian and Laird method). Pooled risk ratios (RRs), 95% CIs, and *P* values were estimated for endpoints in wound healing, all-cause mortality, and amputation using a random-effect statistical model (DerSimonian and Laird method). Heterogeneity was assessed using the *I*^2^ statistic. Subgroup analysis was conducted to investigate the heterogeneity. According to clinical heterogeneity of the wound care model in the control arm, the studies were divided into 2 subgroups. In the community-based model, patients in the control arm received mainly routine wound care by general nurses in community or rural areas, who might not have enough expertise in wound care. In this model, patients might not receive standard care of wound management. By contrast, in the wound center-based model, patients received regular standard outpatient follow-up in the wound center. Sensitivity analysis was conducted by investigating the difference between random and fixed effects model as to effective measures. *P* values ≤.05 were considered statistically significant.

In addition, we tested the hypothesis of noninferiority of TM follow-up for the primary efficacy outcomes. We adopted a Δ=–0.15 as margin of minimum clinically important differences [27,28]. For the primary efficacy outcomes, noninferiority of TM was demonstrated when the lower boundary of the 95% CI was greater than 0.85, which meant that TM could retain at least 85% of the effect of the conventional standard care.

## Results

A total of 12,007 potential studies were identified by the systematic search. Of these, 58 studies were selected for full review. Ultimately, 12 trials met the inclusion criteria, comprising a total of 3913 patients. Of these 5 were cluster RCTs [19-21,29,30], 1 was an RCT [22], and 6 were cohort studies [17,18,27,31-33]. The results of the study selection are shown in Multimedia Appendix 2.

### Study Characteristics

The selected studies [17-22,27,29-33] were published within 15 years. All studies included outpatients or patients in nursing homes. A total of 8 trials [17-19,27,29-32] treated patients with chronic wounds due to different etiologies (mixed wounds), 3 trials [20,22,33] treated patients with diabetic foot ulcers, and 1 study [21] enrolled patients with pressure ulcers. Whereas most studies included any severity of ulcers, one study [20] excluded patients with prior ulcers, which lead to a low proportion of severe ulcers. The method of TM delivery varied between studies. Four studies used tablet or mobile phones programmed with apps, such as Skype and specialized interactive systems [17,20,29,31], whereas other studies used email, telephone, and videoconferencing to facilitate the implementation of TM using specialized systems. However, all methods used included images of wound. The TM consultation specialists included specialized wound care nurses [20,21,32], wound care physicians [17,19,22,27,30,33], podiatrists [20], dermatologists [18,31], and a multidisciplinary team [21]. Wound care varied, particularly in the control group, because of the clinical heterogeneity of TM organization between studies, with 8 of these being community-based models [17-19,21,29-32], and the other 4 wound center-based models [20,22,27,33]. The treatment method and efficacy might have some differences between the two kinds of treatment models. Details of all 12 studies are summarized in Table 1.

### Risk of Bias

The result of assessment of risk of bias is presented in Multimedia Appendices 3-5. For RCTs, because allocation concealment and blinding would not seriously influence the selection of patients and the measurement of outcomes, there was no obvious bias in these two fields. The source of bias for RCTs mainly resulted from uneven baseline characteristics [19,20,29]. For cohort studies, the assessment of risk of bias using ROBINS-I showed that 2 studies [18,32] have moderate risk of bias which demonstrated a sound evidence for a nonrandomized study, whereas 4 studies [17,27,31,33] had serious risk of bias which demonstrated that the studies had some important problems.

**Table 1:** Characteristics of the clinical trials included

Study	Country	Wound etiology	No. of patients		Treatment strategy^c^		Control arm treatment location	Follow-up (months)
			TM	Control	TM	Control		
Smith-Strøm et al [20]	Norway	Diabetic foot ulcer	94	88	Via a web-based ulcer record and phone at least weekly; and outpatient consultation every 6 weeks	Outpatient consultation every second week	University hospital outpatient clinic	12
Stern et al [21]	Canada	Pressure ulcer	93	131	MDT consultation by email, telephone, or video	Usual care	Community	12
Vowden and Vowden [29]	UK	Any etiology	17	9	Consultation by wound-assessment form and images weekly	Home nursing	Nursing home	6
Terry et al [19]	USA	Various etiology^d^	62	98	Consultation by images weekly	Usual care	Home	16
Santamaria et al [30]	Australia	Any etiology	50	43	Consultation by images and measurements every 2 weeks	Care from local wound care clinician	Local clinic	12
Rasmussen et al [22]	Denmark	Diabetic foot ulcer	193	181	Two consultations by telephone or online written consultations and one outpatient consultation cycle	Three outpatient consultation cycle	Wound center	12
Le Goff-Pronost et al [31]	France	Any etiology	77	39	Via videoconference and photos once a week	Primary care	Home	9
Gamus et al [27]	Israel	Any etiology	277	373	Via videoconference	Outpatient clinic	Central clinic	35
Wickström et al [17]	Sweden	Any etiology	100	1888	Video consultation	Primary care	Home	24
Bergersen et al [32]	Norway	Any etiology	32	21	Via wound support network every 4 weeks	Primary home care	Home	3
Zarchi et al [18]	Denmark	Any etiology^e^	50	40	Via a web-based program at a minimum of every second week	Home-care nursing	Home	12
Wilbright et al [33]	USA	Diabetic foot ulcer	20	120	Via real-time interactive video weekly	Face-to-face consultation	Wound center	3

^a^The TM arm received primary care in collaboration with specialists in wound center; patients in the control arm received follow-up by community nurses; in addition, patients in the wound center-based model received treatment at wound center.

^b^TM: telemedicine.

^c^MDT: multidisciplinary teams (comprising 2 enterostomal nurses and 1 certified wound-care nurse, or hospital-based wound-expert team)

^d^Nonhealing surgical wound, stasis ulcer, pressure ulcer.

^e^Surgical wounds, pressure ulcers, and cancer wounds excluded.

### Wound Healing

**Figure 1:**
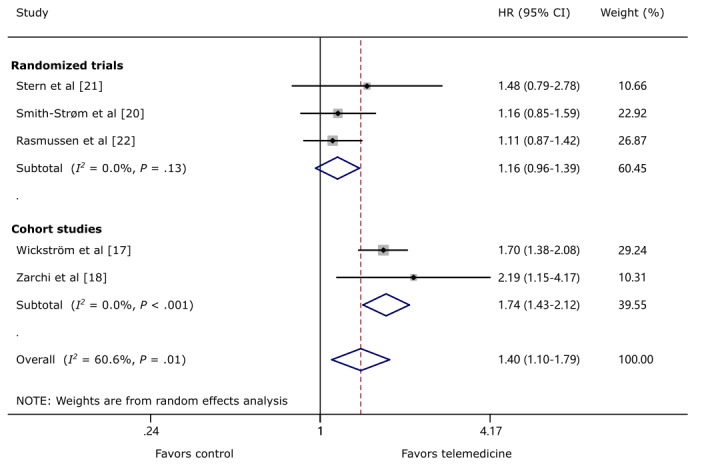
The effect of telemedicine on wound healing. HR: hazard ratio.

Five studies [17,18,20-22] reported data on time to healing. Four studies [18,20-22] directly reported the effect measures HR and CI; however, HR was estimated from the Kaplan–Meier curve in the other remaining study [17]. Overall, TM appeared to demonstrate significant improvement in wound healing (HR 1.40, 95% CI 1.10-1.79; *P*=.01; *I*^2^=60.6%; Figure 1). *I*^2^ statistic of HR was 60.6% (*P*=.04), consistent with moderate heterogeneity of the analysis. In analysis stratified by study design, there was a trend in favor of TM in RCTs (HR 1.16, 95% CI 0.96-1.39; *P*=.13; *I*^2^=0.0%). The lower boundary of HR in RCTs was higher than 0.85, and the criteria of noninferiority of TM were met for wound healing. In addition, statistical difference was demonstrated in cohort studies demonstrating that TM had a decreased risk of allowing unhealed ulcers (HR 1.74, 95% CI 1.43-2.12; *P*<.001; *I*^2^=0.0%). We conducted a subgroup analysis of RCTs to investigate whether a different model of TM organization would result in different clinical effects. The studies that adopted the wound center–based model had a pooled HR of 1.13 (95% CI 0.93-1.37; *P*=.22; *I*^2^=0.0%), and the criteria of noninferiority of TM were met (Multimedia Appendix 6). The 2 cohort studies were both community-based models.

**Figure 2:**
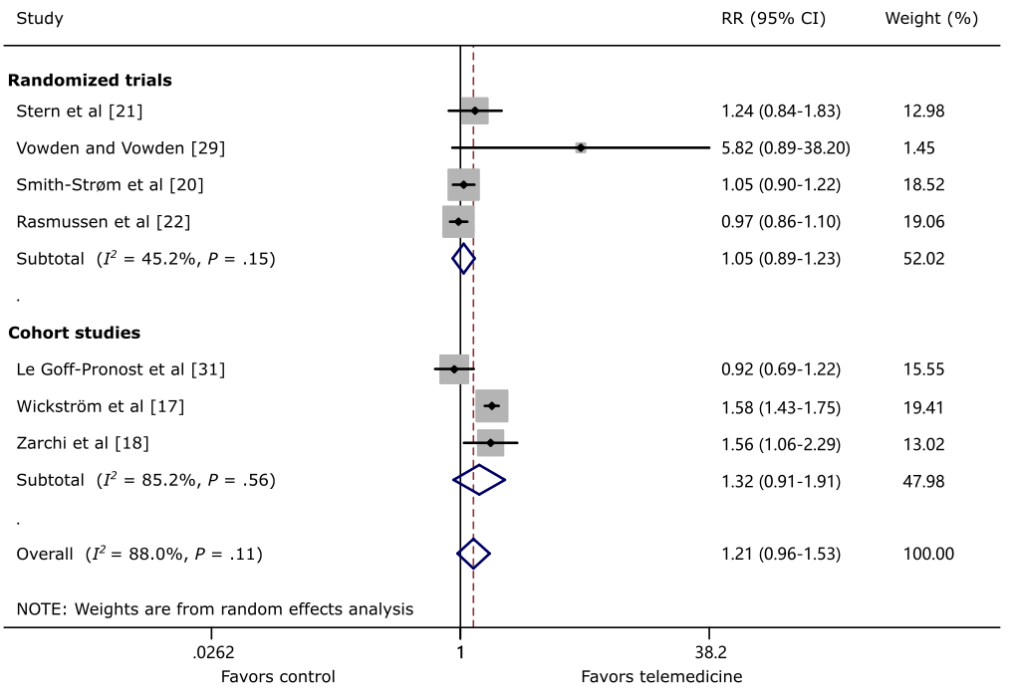
The effect of telemedicine on wound healing around 1 year. RR: risk ratio.

Eight studies [17-22,29,31] reported a wound healing rate of around 1 year, including 5 RCTs and 3 cohort studies. One RCT [19] was not included in the quantitative synthesis because of uneven distribution of severity of wounds among groups. The pooled data of RCTs comparing TM and control showed no statistically significant difference in wound healing (RR 1.21, 95% CI 0.96-1.53; *P*=.11; *I*^2^=88.0%; Figure 2). This finding is consistent in RCTs (RR 1.05, 95% CI 0.89-1.23; *P*=.15; *I*^2^=45.2%) and cohort studies (RR 1.32, 95% CI 0.91-1.91; *P*=.56; *I*^2^=85.2%). However, these statistical trends were in favor of TM, and the criteria of noninferiority of TM were met.

**Figure 3:**
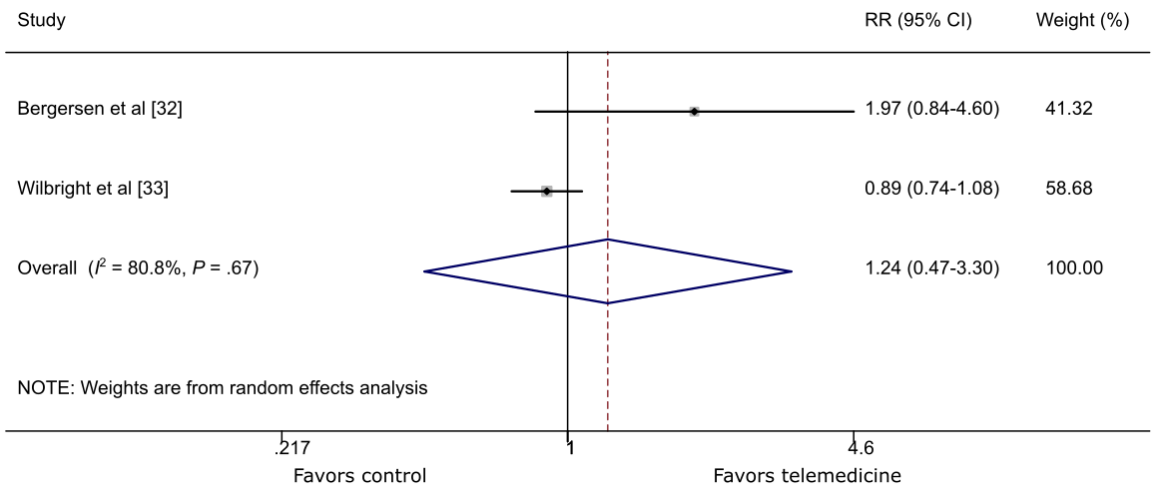
The effect of telemedicine on wound healing around 3 months. RR: risk ratio.

Two studies [32,33] reported wound healing at 3 months. Although there was a trend in favor of the TM group, no statistically significant difference between TM and control on wound healing at 3 months was revealed (RR 1.24, 95% CI 0.47-3.3; *P*=.67; *I*^2^=80.8%; Figure 3).

One study [30] revealed a positive healing rate of 6.8% per week, whereas controls had a negative rate of –4.9% per week (*P*=.01). In this study, no exact number of healed wounds and time to healing were provided, and therefore, the study was not considered in the quantitative analysis.

Another study [27] adopted positive outcomes (indicating at least 50% ulcer closure) as the primary outcome, so it was not included in the quantitative analysis. In this study, equality of TM and face-to-face methods was assessed using 2 one-sided noninferiority tests (WinPepi and the Westlake–Schuirmann method), and the noninferiority of TM was demonstrated within the Δ=0.15 range limits and 80% statistical power.

In the trial with uneven distribution of severity and type of wounds among groups [19], the TM group had significantly larger wound size (*P*=.03) and more severe pressure ulcers. Although wounds in the TM group took longer time to heal and required more resources, it seems as though a greater change in size for pressure ulcers and other wounds occurred.

### All-Cause Mortality

**Figure 4:**
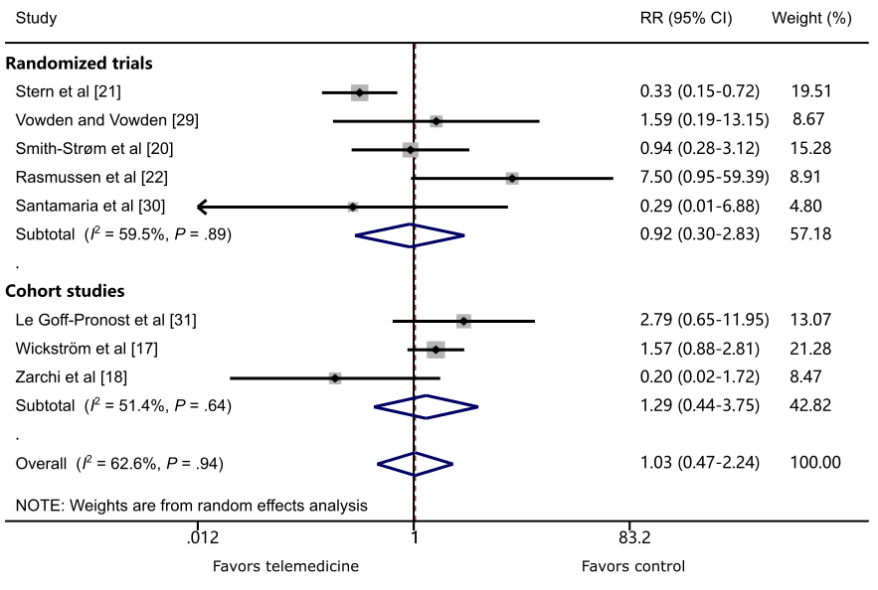
The effect of telemedicine on all-cause mortality. RR: risk ratio.

Eight studies [17,18,20-22,29-31] reported all-cause mortality around 1 year. Pooled data revealed no significant difference in mortality rate between the TM and control groups (RR 1.03, 95% CI 0.47-2.24; *P*=.94; *I*^2^=62.6%; Figure 4). This finding is consistent in RCTs (RR 0.92, 95% CI 0.30-2.83; *P*=.89; *I*^2^=59.5%) and cohort studies (RR 1.29, 95% CI 0.44-3.75; *P*=.64; *I*^2^=51.4%). Subgroup analysis of RCTs reveals there was a statistically significant decreased risk of all-cause mortality in patients receiving TM in the community-based model (RR 0.39, 95% CI 0.19-0.79; *P*=.01; *I*^2^=0.0%); however, no statistical difference in mortality in the wound center–based model was demonstrated (RR 2.25, 95% CI 0.28-18.13; *P*=.45; *I*^2^=67.9%; Multimedia Appendix 7).

### Amputation

**Figure 5:**
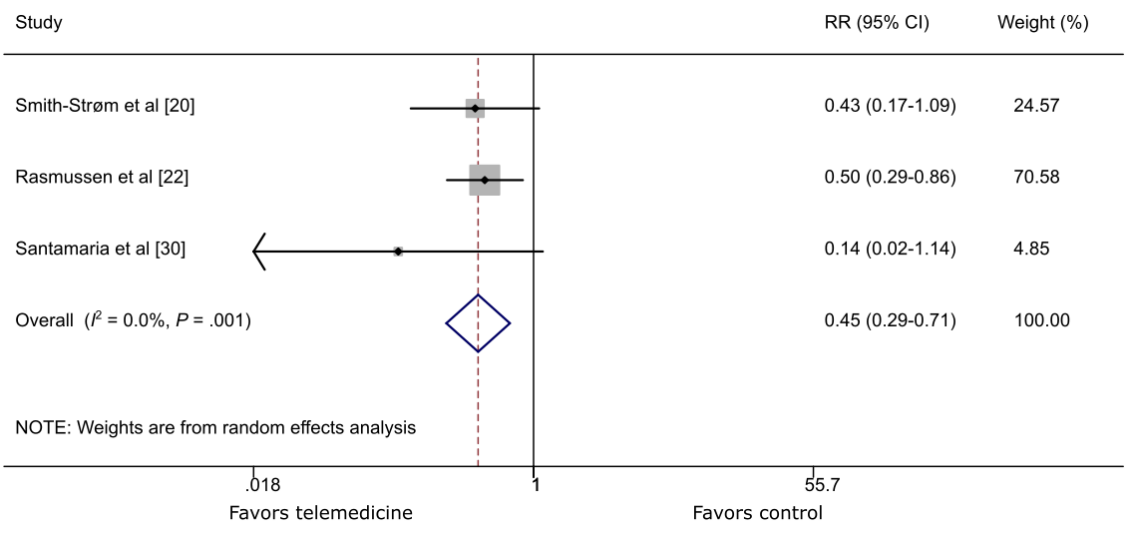
The effect of telemedicine on amputation. RR: risk ratio.

Three RCTs [20,22,30] reported amputation around 1 year. There was a statistically decreased risk of amputation in patients receiving TM follow-up care compared with conventional care (RR 0.45, 95% CI 0.29-0.71; *P*=.001; *I*^2^=0.0%; Figure 5).

### Number of Consultations

Three studies reported on the number of consultations. One RCT [20] revealed that mean consultations at the outpatient clinic per month in the TM group were not statistically different from conventional standard care of wounds (2.0 [SD 1.9] vs 2.5 [SD 3.0]). One cohort study also demonstrated no significant difference between TM and face-to-face follow-up (7.74 [SD 6.79] vs 9.18 [11.05]; *P*=.20) [27]. These two studies adopted a wound center-based model. Another cohort study [32], which adopted a community-based model, showed that the TM group had fewer (mean) appointments at the hospital compared with the control group (1.37 vs 2.33, *P*<.001).

### Patient Satisfaction

One study [20] evaluated patient satisfaction using the Generic Short Patient Experiences Questionnaire, and revealed that there was no statistically significant difference between the two groups (mean difference 0.07, 95% CI –0.10 to 0.24).

### Economic Evaluation

A total of 4 studies [19,21,22,32] mentioned a cost analysis of TM intervention versus control. A detailed economic analysis of 1 study [22] was published in another article [34], which found that the TM follow-up was US $2300 less per patient compared with standard care; however, the difference was not statistically significant (*P*=.42). Another 2 studies [21,32] also revealed reduced cost. One study [19] revealed a higher mean total cost per patient because of larger and more severe ulcers in the TM group.

### Sensitivity Analysis

The sensitivity analysis is presented in Multimedia Appendix 8. All the efficacy outcomes were consistent between random and fixed models, except for wound healing around 1 year of cohort studies. We adopted the most conservative efficacy outcome (RR) using the random model, which still met the criteria of noninferiority of TM.

## Discussion

### Principal Results

In this review, we included 6 RCTs and 6 cohort studies comprising 3913 patients to evaluate the effect of TM in chronic wound management. We adopted both HR and RR to evaluate the effect of TM. In RCTs, we observed no significant differences in the primary clinical outcome efficacies HR and RR around 1 year, and noninferiority criteria were met. In cohort studies, the outcome efficacy HR was in favor of TM, whereas the efficacy RR around 1 year was not significantly different between TM and conventional standard of care of chronic wounds. Overall, these results showed that TM was noninferior to conventional standard of care. In terms of mortality, TM was not significantly different from control in both RCTs and cohort studies. A decreased risk of amputation was observed in patients receiving TM. A few studies performed qualitative analysis on the number of consultations, patient satisfaction, and economic evaluation with the results showing that TM was not worse than conventional standard of care of chronic wounds. Therefore, TM seems to be a safe and effective method in the management of chronic wounds.

We carefully observed the difference of primary outcome between RCTs and cohort studies and found that the enrolled studies in RCTs were mainly wound center-based models, whereas those in cohort studies were all community-based models. Therefore, we speculate that the organization model may have a great influence on the effect of TM on wound healing; in particular, the community-based model may benefit from the implementation of TM. A possible explanation may be that in the community-based model, TM allowed patients in remote and rural settings easier access to multidisciplinary management which has been demonstrated to be an effective and efficient way of chronic wound management [35,36].

Subgroup analysis of RCTs suggested that in a community-based model, patients in the TM group have a decreased risk of mortality. Both the positive primary outcome in cohort studies and the decreased mortality are in favor of TM in the community-based model. This demonstrates that it is promising to take advantage of TM in community or remote rural areas.

### Comparison With Prior Studies

To our knowledge, this review included the largest number of patients with different types of wounds. The results of this review coincide with the systematic review by Tchero et al [16], which included 2 studies to investigate the effectiveness of TM in diabetic foot ulcer management. The authors found that patients in the TM and control groups had similar healing time (43 vs 45 days; *P*=.83) as well as similar ulcer healing rate (odds ratio 0.86, 95% CI 0.57-1.33; *P*=.53). A 10-year study of 5795 patients in France also provided an example of how TM might be of benefit in wound care [37].

Although similar mortality rates were revealed between TM and conventional care of chronic wound, the result of a well-designed RCT [22] revealed a higher mortality in the TM group (HR 8.68, 95% CI 6.93-10.88; *P*=.0001). In the authors’ opinion, the dependence of TM on secondhand information from a nurse could have caused some vital information to be missed. Therefore, it is worth noting that the severity of wounds and other commodities should be taken into consideration when health care participants are considering the use of TM to manage chronic wounds.

For the first time, we learned that the difference between the community- and wound center–based wound management models might seriously influence the effect of TM. A subgroup analysis was conducted to clarify the difference. Differing results between these two models indicate that it is prudent to understand the management model before interpreting the studies on TM in chronic wound management.

### Limitations

This study has several limitations. First, we only searched 3 databases and did not include non-English literature. Although we tried to identify articles from reference lists of other reviews, it is possible that some studies in other databases or published in other language were overlooked. Second, we included RCTs and interventional cohort studies in the analysis, and thus, a potential source of bias might be introduced. Third, several studies [29,32,33] were deemed to be too small to be of statistical significance. Furthermore, in 1 trial [21], a large proportion of censored participants (107/201, 53%) could reduce the effective sample size, thus potentially introducing bias. Fourth, there were obvious variations between studies regarding number of participants, wound etiologies and degree of severity, and implementation of TM, such as video consultation, telephone, email, and picture transmission. Finally, with the ascent of information age, there has been a growing interest in TM. Researchers might tend to publish positive results. All these variations might contribute to bias.

### Implications for Future Studies and Clinical Practices

First, for RCTs, although blinding of outcome measurement would not seriously influence the results, nonblinding of participants might bias the effect. In future studies, more importance should be assigned to the blinding of participants and health care providers. For example, all participants can receive treatment/suggestions via TM, but the information would not be sent to wound center specialists. In this way, performance bias could be reduced to a minimum.

Second, subgroup analysis indicates that TM in the community-based model is superior to standard primary care of chronic wounds by presenting with better outcomes and less mortality. Therefore, it is promising to take advantage of TM in community or remote rural areas. However, the number of studies in this aspect is limited and most studies are cohort studies. Thus, in future studies, well-designed, large-scale RCTs should be performed to verify the effect of TM in the community-based model.

Third, subgroup analysis indicates that TM in the wound center-based model is similar to standard of care of chronic wounds. Therefore, it is necessary to investigate whether TM can have better performance in other aspects. Only a few studies showed that TM was not worse than conventional standard of care regarding number of consultations, patient satisfaction, and economic evaluation. Thus, in future studies, these aspects can be included in the design of trials to investigate the effect of TM.

Finally, results in this systematic review and meta-analysis also shed some light on clinical practices. If health care practitioners would like to use TM to improve wound healing, they do not have to worry about delayed wound healing. For those patients who lived far away from wound center, TM can provide appropriate wound management.

### Conclusions

TM is noninferior to conventional standard care of chronic wounds. TM might be a prosperous method for improving outcomes of patients living in remote or rural areas. However, owing to the relatively low quality of evidence, well-designed and adequately powered RCTs are further needed to confirm the role of TM.

## Appendix

Multimedia Appendix 1 Search strategy.

Multimedia Appendix 2 Flow chart for study selection.

Multimedia Appendix 3 Risk of bias graph of RCTs.

Multimedia Appendix 4 Risk of bias summary of RCTs.

Multimedia Appendix 5 Risk of bias of cohort studies by the use of ROBINS-I.

Multimedia Appendix 6 Subgroup analysis of telemedicine on wound healing.

Multimedia Appendix 7 Subgroup analysis of telemedicine on all-cause mortality.

Multimedia Appendix 8 Results of sensitivity analysis.

## Abbreviations

HR: hazard ratio
RCT: randomized controlled trials
RR: risk ratio
TM: telemedicine
