# Supplementary material for: Telemedicine in Chronic Wound Management: Systematic Review And Meta-Analysis
Source: JMIR Mhealth Uhealth. 2020 Jun 25;8(6):e15574. doi: 10.2196/15574 (PMC7381084; doi:10.2196/15574)
Supplement: Multimedia Appendix 1 [file mhealth_v8i6e15574_app1.pdf]

Multimedia Appendix 1. Search strategy

Database	Search Syntax
Pubmed	(wound OR ulcer OR diabetic foot OR diabetic foot ulcer OR arterial ulcer OR venous ulcer OR stasis ulcer OR varicose ulcer OR pressure ulcer OR "Wounds and Injuries"[Mesh]) AND (telemedicine OR telehealth OR remote consultation OR mobile health OR mhealth OR ehealth OR telephone OR video OR internet OR "Telemedicine"[Mesh])
Embase	(wound OR ulcer OR 'chronic wound'/exp OR diabetic foot OR diabetic foot ulcer OR arterial ulcer OR venous ulcer OR stasis ulcer OR varicose ulcer OR pressure ulcer) AND ('telemedicine'/exp OR telemedicine OR 'telehealth'/exp OR telehealth OR remote consultation OR mobile health OR mhealth OR ehealth OR telephone OR video OR internet)
CENTRAL	(wound OR ulcer OR diabetic foot OR diabetic foot ulcer OR arterial ulcer OR venous ulcer OR stasis ulcer OR varicose ulcer OR pressure ulcer) AND (telemedicine OR telehealth OR remote consultation OR mobile health OR mhealth OR ehealth OR telephone OR video OR internet OR MeSH term telemedicine)
