# Supplementary figures and images for: Telemedicine in Chronic Wound Management: Systematic Review And Meta-Analysis
Source: JMIR Mhealth Uhealth. 2020 Jun 25;8(6):e15574. doi: 10.2196/15574 (PMC7381084; doi:10.2196/15574)

Identification

Screening

Eligibility

Included

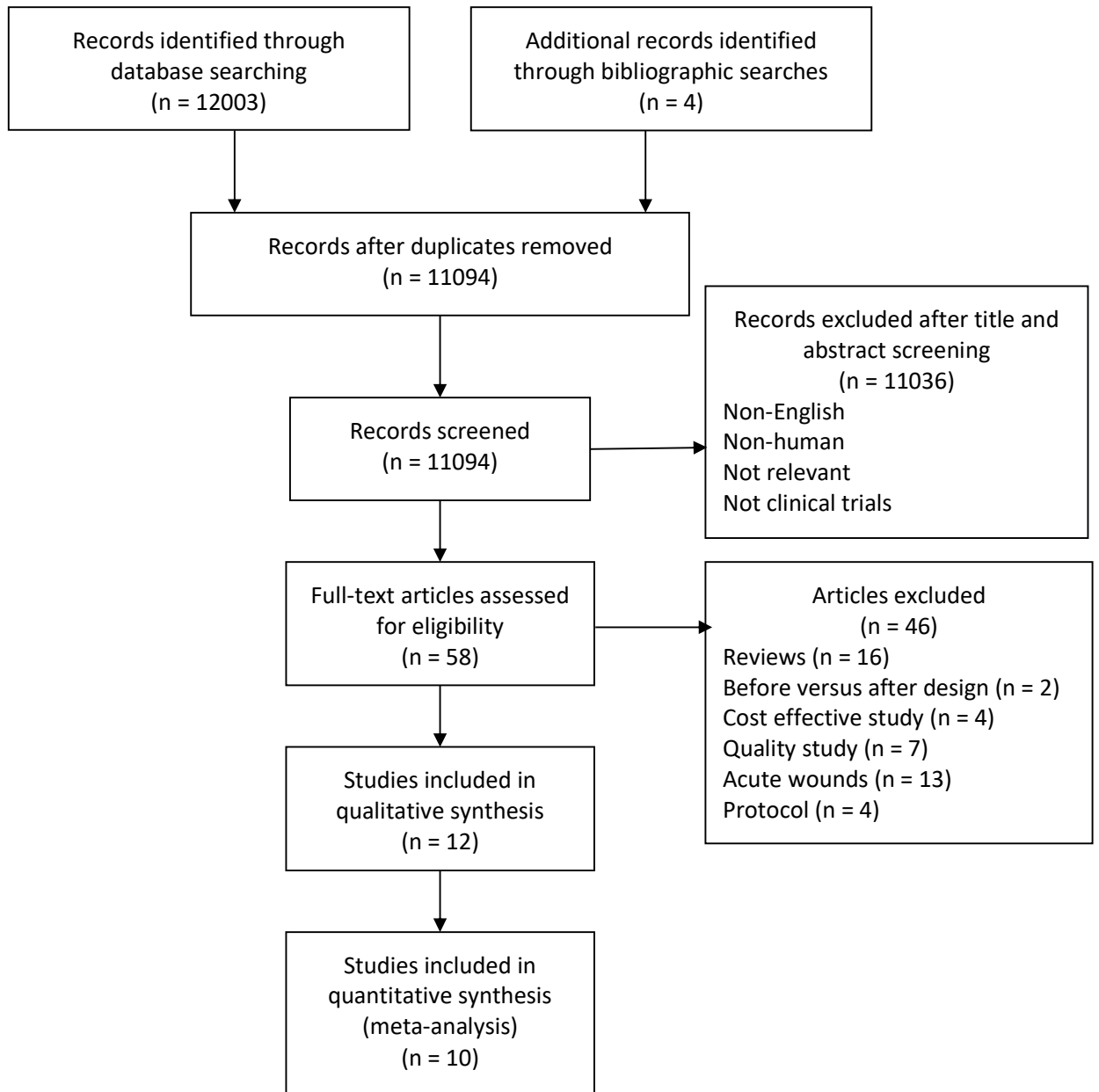

Supplement: Multimedia Appendix 2 [file mhealth_v8i6e15574_app2.pdf]

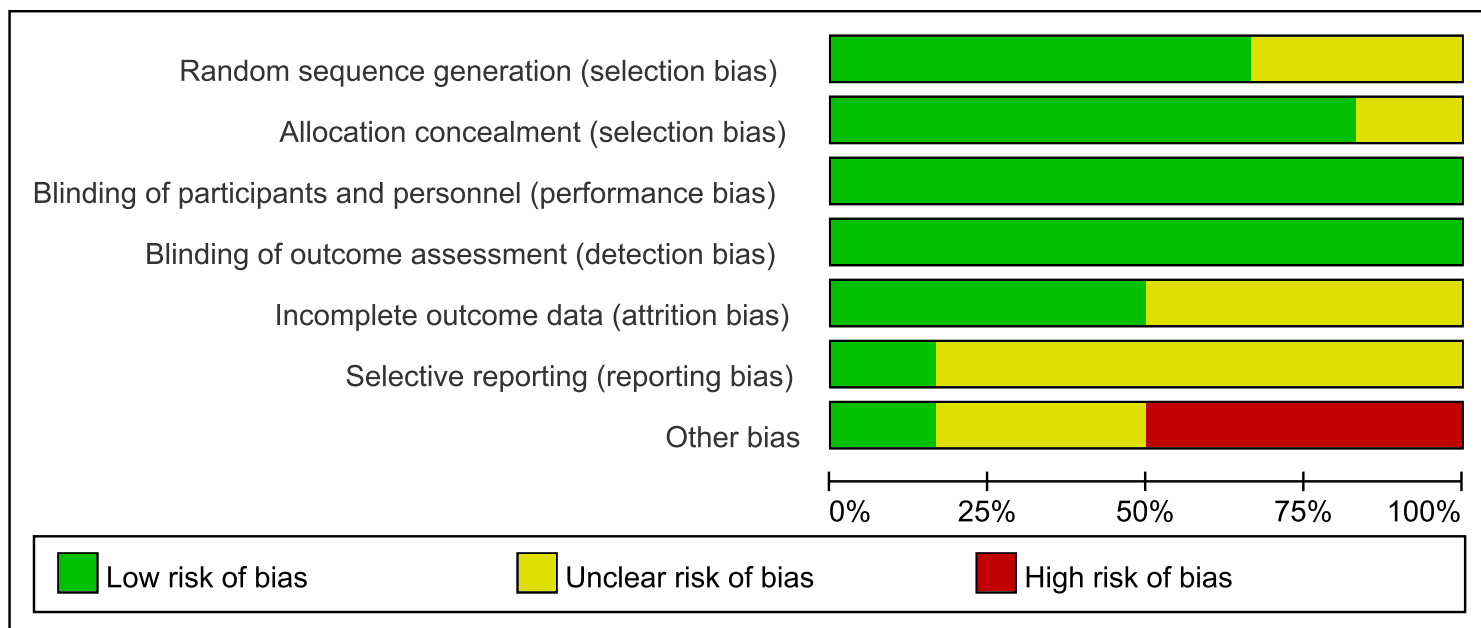

Supplement: Multimedia Appendix 3 [file mhealth_v8i6e15574_app3.pdf]

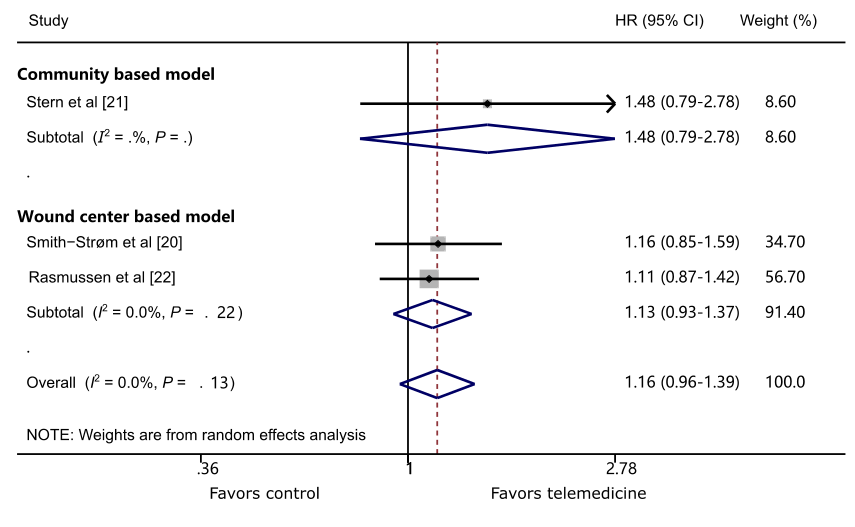

Supplement: Multimedia Appendix 6 [file mhealth_v8i6e15574_app6.PNG]

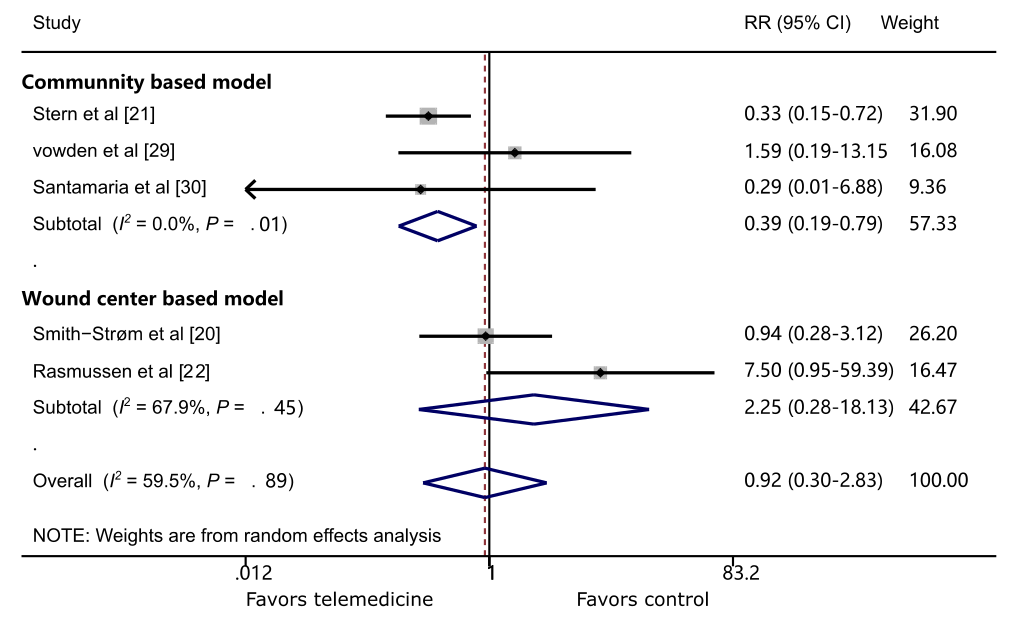

Supplement: Multimedia Appendix 7 [file mhealth_v8i6e15574_app7.PNG]
