# Supplementary material for: Telemedicine in Chronic Wound Management: Systematic Review And Meta-Analysis
Source: JMIR Mhealth Uhealth. 2020 Jun 25;8(6):e15574. doi: 10.2196/15574 (PMC7381084; doi:10.2196/15574)
Supplement: Multimedia Appendix 4 [file mhealth_v8i6e15574_app4.pdf]

	Random sequence generation (selection bias)	Allocation concealment (selection bias)	Blinding of participants and personnel (performance bias)	Blinding of outcome assessment (detection bias)	Incomplete outcome data (attrition bias)	Selective reporting (reporting bias)	Other bias
Rasmussen 2015	+	+	+	+	+	+	+
Santamaria 2004	?	?	+	+	?	?	?
Smith-Strom 2018	+	+	+	+	+	?	−
Stern 2014	+	+	+	+	+	?	?
Terry 2009	?	+	+	+	?	?	−
Vowden 2013	+	+	+	+	?	?	−
