# Supplementary material for: Telemedicine in Chronic Wound Management: Systematic Review And Meta-Analysis
Source: JMIR Mhealth Uhealth. 2020 Jun 25;8(6):e15574. doi: 10.2196/15574 (PMC7381084; doi:10.2196/15574)
Supplement: Multimedia Appendix 5 [file mhealth_v8i6e15574_app5.pdf]

Multimedia Appendix 5_ Risk of bias of cohort studies by the use of ROBINS-I

	**Bias due to confounding**	**Selection bias**	**Bias in classification of interventions**	**Bias due to deviations from intended interventions**	**Bias due to missing data**	**Bias in measurement of outcomes**	**Bias in selection of the reported result**	**Overall bias**
Bergersen 2016	Moderate	Low	Low	Low	Moderate	Low	Moderate	Moderate
Gamus 2019	Moderate	Serious	Low	Moderate	Serious	Low	Low	Serious
Le Goff-Pronost 2018	Serious	Low	Low	Low	Moderate	Low	Moderate	Serious
Wickstrom 2018	Serious	Low	Low	Low	Moderate	Low	Moderate	Serious
wilbright 2004	Serious	Low	Low	Low	Moderate	Low	Moderate	Serious
Zarchi 2015	Moderate	Low	Low	Low	Moderate	Low	Low	Moderate
