# Supplementary material for: Telemedicine in Chronic Wound Management: Systematic Review And Meta-Analysis
Source: JMIR Mhealth Uhealth. 2020 Jun 25;8(6):e15574. doi: 10.2196/15574 (PMC7381084; doi:10.2196/15574)
Supplement: Multimedia Appendix 8 [file mhealth_v8i6e15574_app8.pdf]

Multimedia Appendix 8.1_Sensitivity analysis of RCTs: random model vs. fixed model

		Wound healing	Wound healing around 1 year	Wound healing around 3 months	mortality	amputation
HR	Random model	1.16 (0.96, 1.39)	-	-	-	-
	Fixed model	1.16 (0.96, 1.39)	-	-	-	-
RR	Random model	-	1.02 (0.83, 1.24)	-	0.83 (0.33, 2.09)	0.45 (0.29, 0.72)#
	Fixed model	-	1.01 (0.91, 1.12)	-	0.86 (0.58, 1.27)	0.44 (0.28, 0.69)#
OR	Random model	-	1.06 (0.60, 1.89)	-	0.80 (0.28, 2.24)	0.41 (0.25, 0.68)#
	Fixed model	-	1.03 (0.77, 1.38)	-	0.85 (0.54, 1.34)	0.40 (0.24, 0.66)#
RD	Random model	-	0.05 (-0.08, 0.17)	-	-0.02 (-0.09, 0.05)	-0.08 (-0.13, -0.04)#
	Fixed model	-	0.01 (-0.05, 0.06)	-	0.01 (-0.05, 0.03)	-0.08 (-0.13, -0.04)#

Random model: D-L method; Fixed model: I-V method (HR), M-H method (RR, OR RD);

#, P＜0.05

Multimedia Appendix 8.2_Sensitivity analysis of cohort studies: random model vs. fixed model

		Wound healing	Wound healing around 1 year	Wound healing around 3 months	mortality	amputation
HR	Random model	1.74 (1.43, 2.12)#	-	-	-	-
	Fixed model	1.74 (1.43, 2.12)#	-	-	-	-
RR	Random model	-	1.32 (0.91, 1.91)	1.29 (0.47, 3.30)	2.25 (0.28, 18.13)	-
	Fixed model	-	1.43 (1.29, 1.58)#	1.06 (0.84, 1.34)	2.03 (0.78, 5.25)	-
OR	Random model	-	2.17 (1.20, 6.80)#	0.95 (0.10, 8.77)	2.30 (0.28, 19.07)	-
	Fixed model	-	2.76 (1.91, 4.01)#	1.30 (0.52, 3.28)	2.08 (0.78, 5.59)	-
RD	Random model	-	0.17 (-0.05, 0.39)	0.05 (-0.29, 0.39)	0.03 (-0.01, 0.04)	-
	Fixed model	-	0.23 (0.16, 0.30)#	0.04 (-0.10, 0.18)	0.02 (-0.01, 0.05)	-

Random model: D-L method; Fixed model: I-V method (HR), M-H method (RR, OR RD);
#, P＜0.05
